# Chronic venous ulcer resolution and post-thrombotic syndrome improvement after percutaneous mechanical thrombectomy of a 42-year-old deep vein thrombosis

**DOI:** 10.1016/j.jvscit.2022.03.001

**Published:** 2022-03-11

**Authors:** Nicolas J. Mouawad

**Affiliations:** Division of Vascular and Endovascular Surgery, Department of Surgery, McLaren Health System, Bay City, MI

**Keywords:** ClotTriever, Deep vein thrombosis, Post-thrombotic syndrome, Thrombectomy, Venous ulcer

## Abstract

Post-thrombotic syndrome (PTS) is a chronic disease affecting up to one half of patients with deep vein thrombosis. PTS symptoms range in severity, with the worst form involving the development of venous ulcers. In the present report, we have described a patient with PTS with ulceration for >40 years. A percutaneous approach with a mechanical thrombectomy extirpation device was used to remove the chronic thrombus in a single session. At >3 months of follow-up, the PTS symptoms had improved dramatically, and the ulcer had completely healed. We have demonstrated successful removal of long-standing chronic thrombus using the ClotTriever system (Inari Medical, Irvine, CA) and the management of venous ulcers resulting from debilitating PTS.

Each year, 100 to 300/100,000 persons will be diagnosed with deep vein thrombosis (DVT) in the United States.^1^^,^^2^ Despite adequate anticoagulation therapy, 20% to 50% of patients with acute DVT will develop a chronic condition termed post-thrombotic syndrome (PTS), a devastating state that includes the additional symptoms of pruritus, paresthesia, and, in the most severe stage, ulceration.^3^

Approximately 5% of patients with DVT will develop a venous ulcer within 10 years.^4^^,^^5^ For those patients whose ulcers heal, the recurrence rates have ranged from 26% to 69% within 1 year, depending on the treatment and prevention strategies used.^6^^,^^7^ Ulcers are a debilitating consequence of chronic venous hypertension that significantly diminishes patients' quality of life, with nearly 90% of patients with PTS unable to work at 10 years after the diagnosis.^8^ In severe cases, PTS can even lead to limb amputation. A prospective study of the outcomes associated with leg ulcers reported that 4% of patients with ulceration required amputation within 4.5 years.^9^

The development of PTS is primarily linked to increased venous pressure secondary to valve dysfunction or outflow obstruction, usually due to DVT. The current therapeutic options for chronic venous hypertension consist of conservative care, medical therapy, open surgery, or venoplasty and stenting.^10^ Because PTS is not curable, the aim of treatment is to alleviate the symptoms and improve patient quality of life. Most PTS therapies will address only the symptoms of the disease, without removing the underlying occlusion and restoring blood flow, resulting in continued progression of the syndrome. Conservative treatments will often be ineffective, and open surgical reconstruction can result in complications and risks that could be disproportionately high.^11^

Recent advances in endovascular approaches have expanded the capacity to treat DVT. The ClotTriever System (Inari Medical, Irvine, CA), a catheter-based mechanical thrombectomy device designed to treat DVT, has been successful in removing acute, subacute, and chronic thrombus.^12^, ^13^, ^14^ Although no formal definition has been determined for thrombus age, chronic thrombi have generally been accepted to be >2 months old and essentially resistant to thrombolysis, because microscopically they will be more rich in collagen. In the present report, we have outlined a case of chronic venous hypertension and ongoing ulceration that had persisted for decades after an initial DVT diagnosis in 1979. The patient had been referred for amputation; however, after consultation with our venous thromboembolism program, he was successfully treated using the ClotTriever System. The patient provided written informed consent for the report of his case details and imaging studies.

## Case report

A 66-year-old man with severe PTS had presented to our wound care center for worsening of a longstanding, recurrent venous ulcer on his left lower extremity. The patient reported experiencing PTS sequelae for decades after a DVT diagnosis secondary to trauma. He had been seen previously by multiple other providers and wound centers for treatment of the chronic ulcer, with various episodes of conservative therapies using intermittent Unna boot applications and long-term anticoagulation with warfarin therapy. His relevant medical history included a pulmonary embolism after his DVT and left great saphenous vein ablation. Our focused physical examination noted an ∼39 cm^2^ ulcer at the medial calf that was surrounded by hemosiderin deposition and venous eczematous changes with grade 2 to 3+ edema. The laboratory test results were unremarkable, and the thrombophilia reports were negative. His venous clinical severity score at presentation was 21. A repeat full workup was instituted, and a venous duplex ultrasound with reflux assessment identified an absent great saphenous vein and chronic, hyperechoic thrombus from the distal external iliac vein to the posterior tibial vein.

**Fig 1:**
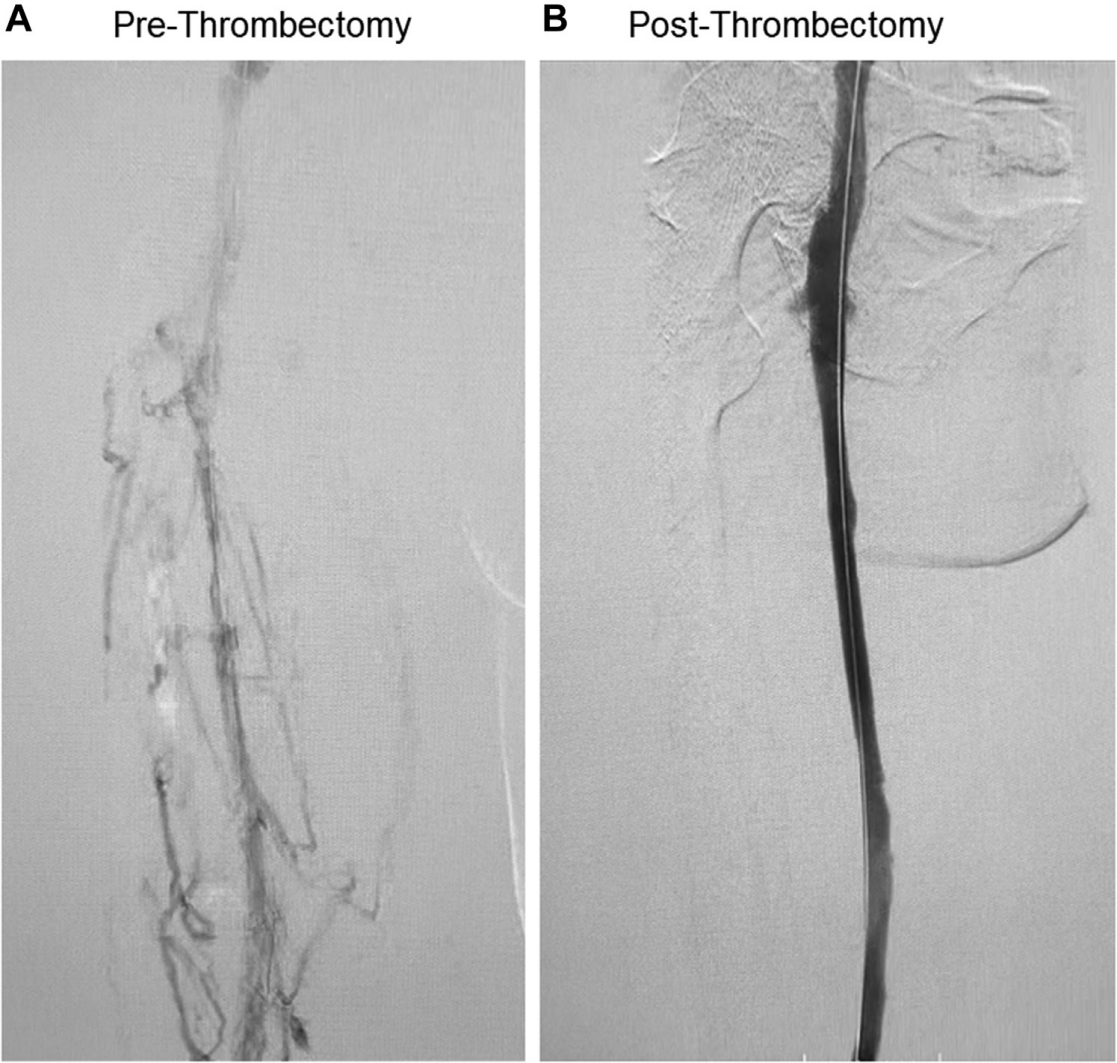
**A,** Prethrombectomy venogram with chronic thrombus and multiple collateral vessels in the femoropopliteal and iliofemoral veins. **B,** Post-thrombectomy venogram of patent vessel after mechanical thrombectomy with the ClotTriever System (Inari Medical, Irvine, CA).

Given the chronicity, mechanical extirpation of the obstructive thrombus using the ClotTriever System (Inari Medical) was considered to increase venous outflow, promote wound closure, and, ultimately, eliminate the option of amputation. The patient was placed prone under conscious sedation. The left popliteal vein was accessed using a modified Seldinger technique with ultrasound guidance. An ascending venogram demonstrated multiple venous collateral vessels with axial luminal filling defects and diseased iliofemoral and iliocaval systems (Fig 1, *A*). Intravascular ultrasound was used to confirm true intravenous positioning and not within a collateral vessel. An activated clotting time of >250 seconds after systemic anticoagulation was confirmed.

**Fig 2:**
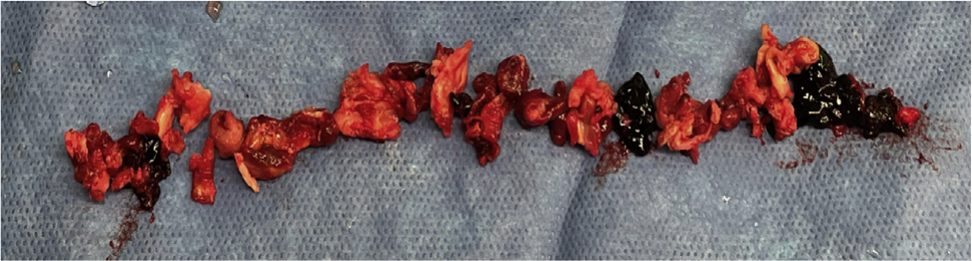
Extirpated thrombus and post-thrombotic collagen with multiple areas of more acute-appearing thrombus.

**Fig 3:**
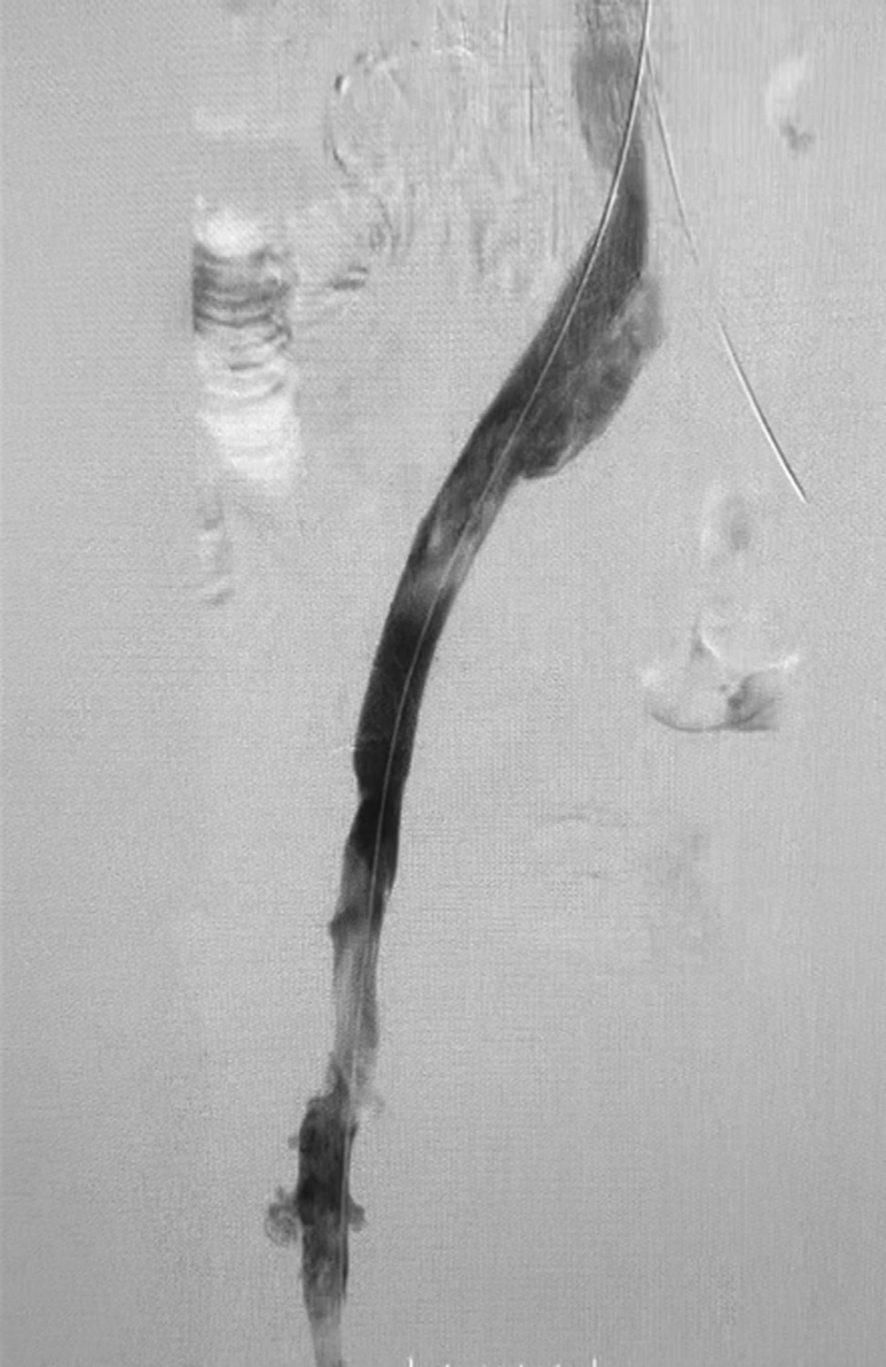
Adjunctive stenting in the iliac vein for post-thrombectomy outflow resolution.

The access site was serially dilated before placement of a 13F ClotTriever sheath. The entire proposed treatment tract underwent venoplasty with an 8-mm balloon in the femoropopliteal segment and a 12-mm balloon within the iliofemoral and iliocaval segments. Seven passes of the ClotTriever device were performed, with the last pass requiring balloon venoplasty to help detach the chronically adherent thrombus from the distal external iliac vein. The ClotTriever catheter, with its expandable nitinol coring element that achieves wall-to-wall apposition, was able to detach and remove significant adherent thrombus burden and chronic occlusions from the venous wall (Fig 2). It also disrupted multiple venous trabeculations and synechiae, restoring vessel patency (Fig 1, *B*). The attached collection bag prevented cephalad embolization. Intravascular ultrasound confirmed left common iliac compression of 67% and left external iliac fibrotic compression of 63% with a cross-sectional area of 29 mm^2^. Adjunctive deep venous stenting was conducted (Fig 3). No lytic agents were used, and the access site was wrapped with a pressure bandage. The patient was discharged home directly from the post-anesthesia care unit with novel anticoagulant and antiplatelet therapy indefinitely.

**Fig 4:**
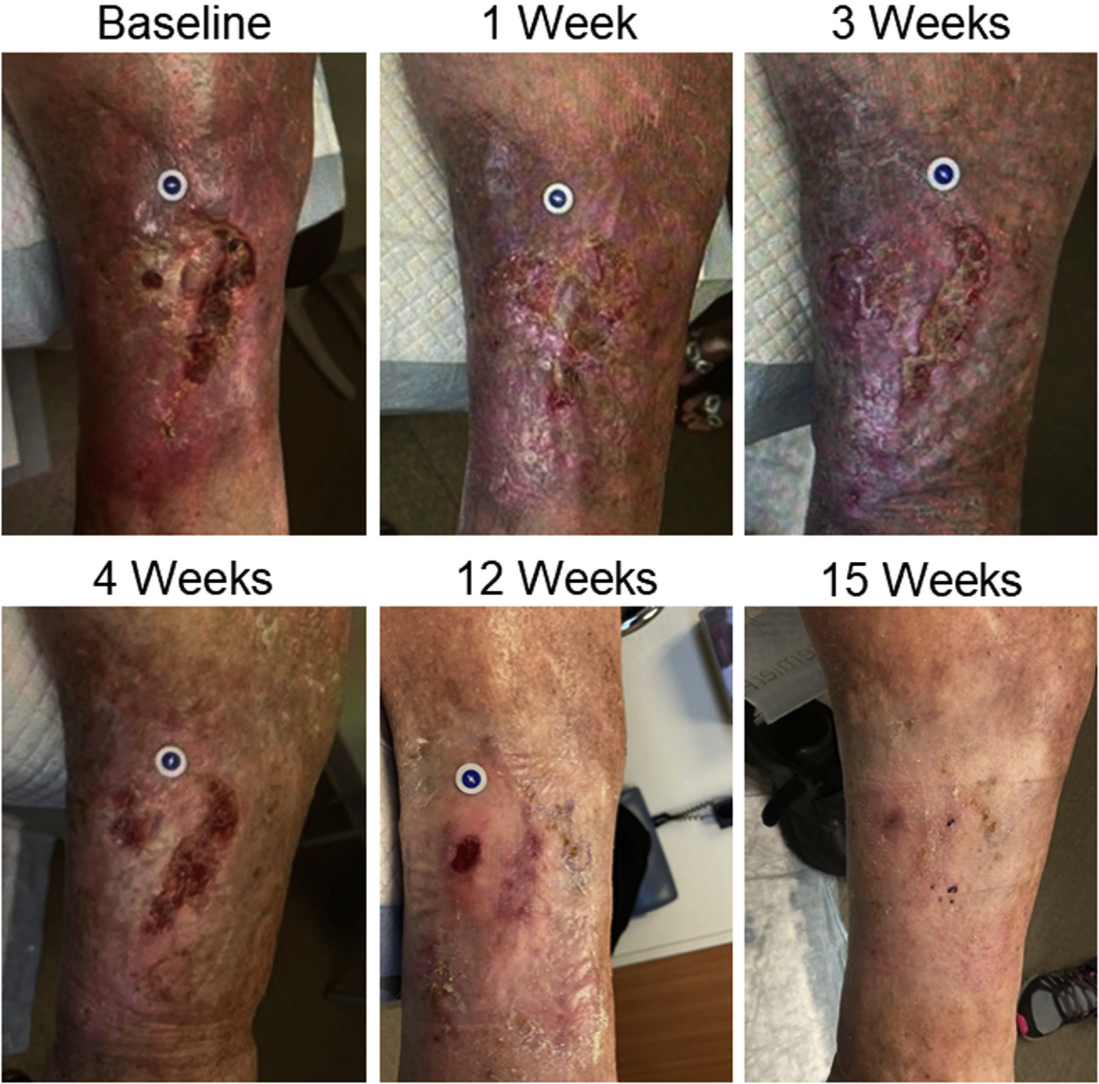
Progression of chronic venous ulcer healing after thrombectomy.

**Fig 5:**
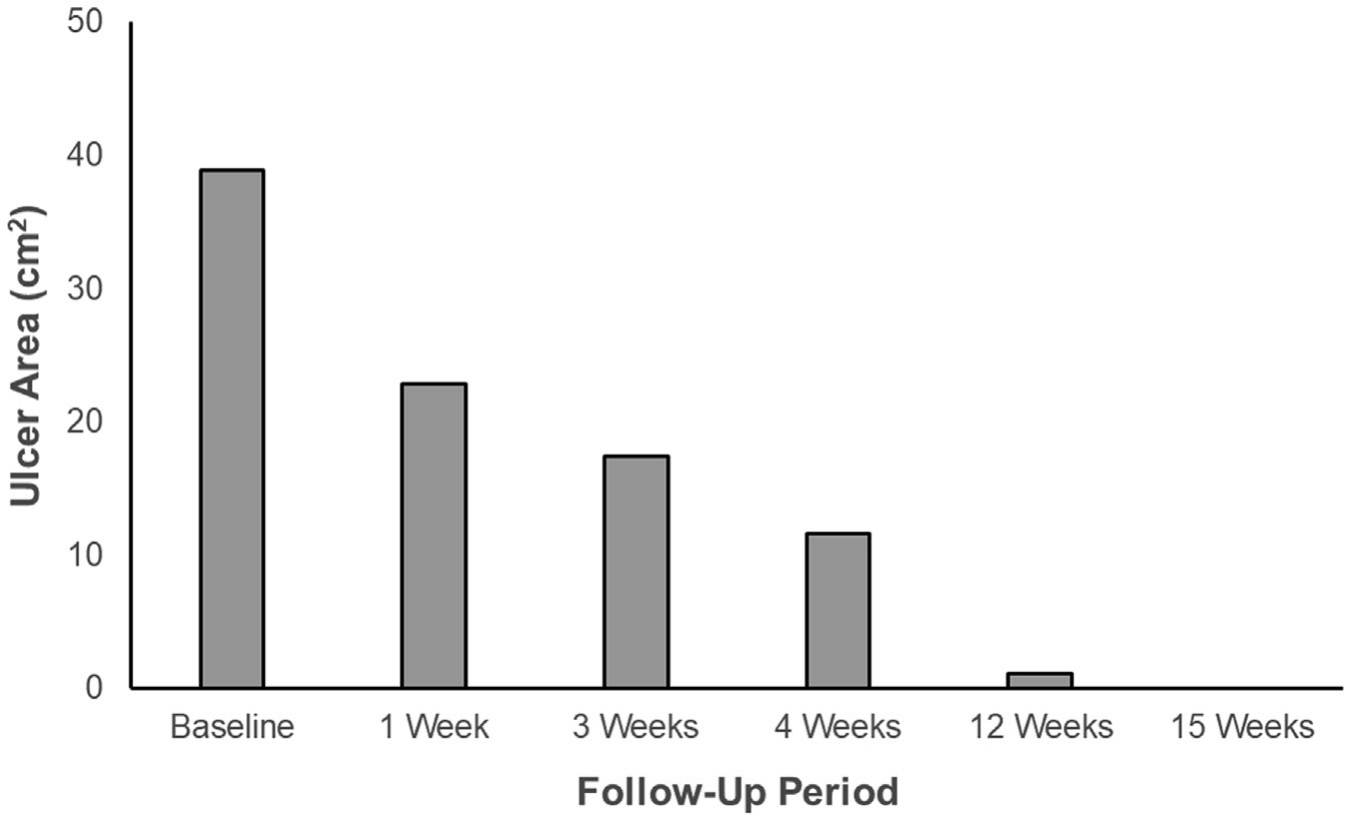
Graph showing reduction in venous ulcer area measured at postprocedure follow-up examinations.

The patient continued weekly visits at our wound center with Unna boot four-layer compression application. Local superficial debridement was performed biweekly. Follow-up ultrasound demonstrated a patent stent and cephalad spontaneous flow. At 12 weeks after the chronic occlusion had been extirpated, the patient's active venous ulcer had almost completely resolved, with the ulcer area decreasing by 97% to only 1 cm^2^. At 15 weeks, the ulcer had completely healed (Figs 4 and 5). The patient reported improvement in additional PTS symptoms, including decreased pain and swelling. At 15 weeks, his venous clinical severity score had decreased to 8.

## Discussion

PTS is a devastating disease, with 200,000 to 500,000 new diagnoses in the United States each year.^1^, ^2^, ^3^ The lack of effective treatment to remove chronic venous occlusions has led to serious clinical outcomes for patients with PTS, including amputation. This chronic state also exacts a steep economic burden. PTS has been associated with a 35% to 45% increase in medical and total costs to patients.^15^ It has also been estimated that PTS is responsible for an annual loss of ∼2 million working days in the United States.^16^

Most available therapies can only treat acute DVT owing to their dependence on thrombolytic agents for clot dissolution.^17^, ^18^, ^19^ However, much of the fibrin in acute thrombus will be replaced by collagen after 14 days, and the thrombus will no longer be degraded effectively using thrombolytic agents.^20^ Nevertheless, these symptom-focused approaches will be ineffective for many patients and result in increasingly severe disease.

Patients with PTS will generally have an obliterated lumen or inner wall venous plane secondary to extensive endoluminal collagen. Traditional open endovenectomy is necessary for luminal gain. In our patient, with aggressive balloon predilatation, we believed that a leading edge of the luminal collagen became exposed by fracturing from the vein wall. Thus, ultimately, the mechanical coring element successfully engaged the detached adherent collagen-laced thrombus. Analysis of our specimen supported this, because we found multiple areas of different colors, indicating a dynamic process of thrombus formation, fibrinolysis, and collagen deposition and thrombi of differing chronicity.

Adjunctive stenting can be necessary, such as in our patient. Therefore, the contribution to overall clinical improvement from thrombus extirpation vs decompressive stenting is difficult to determine. However, we believe strongly that luminal gain and restoration of cephalad inflow via thrombectomy is important. Through an aggressive and established venous thromboembolism program, we suggested using the ClotTriever system as an endovenous mechanical extirpation device owing to its ability to remove chronic longstanding DVT and achieve this objective.^12^^,^^13^

## Conclusions

Although additional studies are warranted, the ClotTriever System appears to be a promising therapy for removing stubborn and chronic thrombus, restoring blood flow, and resolving PTS and venous ulceration. Mechanical extirpation should be considered for the treatment of both acute and chronic DVT and for PTS with chronic ulcers, especially when long-term debility, morbidity, and amputation might be the only alternative.

## References

[1] Vazquez S.R., Kahn S.R. (2012). Advances in the diagnosis and management of postthrombotic syndrome. Best Pract Res Clin Haematol.

[2] Deitelzweig S.B., Johnson B.H., Lin J., Schulman K.L. (2011). Prevalence of clinical venous thromboembolism in the USA: current trends and future projections. Am J Hematol.

[3] Kahn S.R., Comerota A.J., Cushman M., Evans N.S., Ginsberg J.S., Goldenberg N.A. (2014). The postthrombotic syndrome: evidence-based prevention, diagnosis, and treatment strategies: a scientific statement from the American Heart Association. Circulation.

[4] Schulman S., Lindmarker P., Holmstrom M., Larfars G., Carlsson A., Nicol P. (2006). Post-thrombotic syndrome, recurrence, and death 10 years after the first episode of venous thromboembolism treated with warfarin for 6 weeks or 6 months. J Thromb Haemost.

[5] Ashrani A.A., Heit J.A. (2009). Incidence and cost burden of post-thrombotic syndrome. J Thromb Thrombolysis.

[6] Nelson E.A., Adderley U. (2016). Venous leg ulcers. BMJ Clin Evid.

[7] Polak M.W., Siudut J., Plens K., Undas A. (2019). Prothrombotic clot properties can predict venous ulcers in patients following deep vein thrombosis: a cohort study. J Thromb Thrombolysis.

[8] Kahn S.R., Ginsberg J.S. (2004). Relationship between deep venous thrombosis and the postthrombotic syndrome. Arch Intern Med.

[9] Nelzén O., Bergqvist D., Lindhagen A. (1997). Long-term prognosis for patients with chronic leg ulcers: a prospective cohort study. Eur J Vasc Endovasc Surg.

[10] Saha P., Black S., Breen K., Patel A., Modarai B., Smith A. (2016). Contemporary management of acute and chronic deep venous thrombosis. Br Med Bull.

[11] Schleimer K., Barbati M.E., Grommes J., Hoeft K., Toonder I.M., Wittens C.H.A. (2019). Update on diagnosis and treatment strategies in patients with post-thrombotic syndrome due to chronic venous obstruction and role of endovenous recanalization. J Vasc Surg Venous Lymphat Disord.

[12] Abramowitz S. (2021). Effective removal of chronic thrombus with the ClotTriever System: results from the CLOUT Registry. J Vasc Surg Venous Lymphat Disord.

[13] Srivastava A. (2019). Acute thrombolysis-resistant occlusive left femoral and iliac venous thrombosis treated with mechanical thrombectomy via the ClotTriever device. Ann Vasc Surg.

[14] Mouawad N.J. (2020). Effective single-session percutaneous nonpharmacologic mechanical thrombectomy for phlegmasia cerulea dolens. J Vasc Surg Cases Innov Tech.

[15] Guanella R., Ducruet T., Miron M.-J., Roussin A., Desmarais S., Joyal F. (2011). Economic burden and cost determinants of deep vein thrombosis during 2 years following diagnosis: a prospective evaluation. J Thromb Haemost.

[16] Bergan J.J., Schmid-Schönbein G.W., Smith P.D., Nicolaides A.N., Boisseau M.R., Eklof B. (2006). Chronic venous disease. N Engl J Med.

[17] Comerota A.J., Kearon C., Gu C., Julian J.A., Goldhaber S.Z., Kahn S.R. (2019). Endovascular thrombus removal for acute iliofemoral deep vein thrombosis. Circulation.

[18] Enden T., Haig Y., Kløw N., Slagsvold C.E., Sandvik L., Ghanima W. (2012). Long-term outcome after additional catheter-directed thrombolysis versus standard treatment for acute iliofemoral deep vein thrombosis (the CaVenT study): a randomised controlled trial. Lancet.

[19] Garcia M.J., Sterling K.M., Kahn S.R., Comerota A.J., Jaff M.R., Ouriel K. (2020). Ultrasound-accelerated thrombolysis and venoplasty for the treatment of the postthrombotic syndrome: results of the ACCESS PTS study. J Am Heart Assoc.

[20] Yuriditsky E., Narula N., Jacobowitz G.R., Moreira A.L., Maldonado T.S., Horowitz J.M. (2022). Histological assessment of lower extremity deep vein thrombi from patients undergoing percutaneous mechanical thrombectomy. J Vasc Surg Venous Lymphat Disord.

